# Increase in brain activation due to sub-tasks during driving: fMRI study using new MR-compatible driving simulator

**DOI:** 10.1186/s40101-017-0128-8

**Published:** 2017-01-26

**Authors:** Mi-Hyun Choi, Hyung-Sik Kim, Hee-Jeong Yoon, Jung-Chul Lee, Ji-Hye Baek, Jin-Seung Choi, Gye-Rae Tack, Byung-Chan Min, Dae-Woon Lim, Soon-Cheol Chung

**Affiliations:** 1 Department of Biomedical Engineering, Research Institute of Biomedical Engineering, College of Biomedical & Health Science, Konkuk University, Chungju, South Korea; 2 Department of Industrial and Management Engineering, Hanbat National University, Daejeon, South Korea; 3 Department of Information & Communication Engineering, Dongguk University, Seoul, South Korea

**Keywords:** MR-compatible driving simulator, Sub-task (additional task), Driving, Sub-lobar, fMRI

## Abstract

**Background:**

Several studies have used functional magnetic resonance imaging (fMRI) to show that neural activity is associated with driving. fMRI studies have also elucidated the brain responses associated with driving while performing sub-tasks. It is important to note that these studies used computer mouses, trackballs, or joysticks to simulate driving and, thus, were not comparable to real driving situations. In order to overcome these limitations, we used a driving wheel and pedal equipped with an MR-compatible driving simulator (80 km/h). The subjects drove while performing sub-tasks, and we attempted to observe differences in neuronal activation.

**Methods:**

The experiments consisted of three blocks and each block consisted of both a control phase (1 min) and a driving phase (2 min). During the control phase, the drivers were instructed to look at the stop screen and to not perform driving tasks. During the driving phase, the drivers either drove (driving only condition) or drove while performing an additional sub-task (driving with sub-task condition) at 80 km/h.

**Results:**

Compared to when the drivers were focused only on driving, when the drivers drove while performing a sub-task, the number of activation voxels greatly decreased in the parietal area, which is responsible for spatial perception. Task-performing areas, such as the inferior frontal gyrus and the superior temporal gyrus, showed increased activation. Performing a sub-task simultaneously while driving had affected the driver’s driving. The cingulate gyrus and the sub-lobar region (lentiform nucleus, caudate, insula, and thalamus), which are responsible for error monitoring and control of unnecessary movements (e.g., wheel and pedal movements), showed increased activation during driving with sub-task condition compared to driving only condition.

**Conclusions:**

Unlike simple driving simulators (joysticks, computer mouses, or trackballs) used in previous research, the addition of a driving wheel and pedals (accelerator and brake) to the driving simulator used in this study closely represents real driving. Thus, the number of processed movements was increased, which led to an increased number of unnecessary movements that needed to be controlled. This in turn increased activation in the corresponding brain regions.

## Background

Driving is a complex multitasking activity that involves perception, attention, decision-making, sensory, motor, and higher-level cognitive components [1, 2]. Recent studies on complex multitasking (driving)-related neural correlates have used functional magnetic resonance imaging (fMRI) to study its neurophysiological aspects [3–17]. In previous studies, driving was simulated by using a joystick, computer mouse, or a trackball. They revealed the activation of the following regions: the parietal lobe and precuneus region (spatial perception), the precentral gyrus and frontal eye field regions (motor response, arm and eye movement) [5, 12], the supplementary motor area (SMA) and cerebellum region (motor control and action planning) [3, 6, 12, 14–16], and the cingulate gyrus region (attention and error monitoring) [3, 5, 12, 14].

Recently, the increase in electronic device use has resulted in the performance of frequent sub-tasks during driving. Sub-tasks can be defined as radio tuning, dialing a cell phone, eating, or carrying on a conversation. These sub-tasks are reported to decrease driving performance (brake response time, tracking performance, speed control, car following, and lane keeping) measured using physiological assessments [1, 2, 7, 11, 15]. Safe driving requires the ability to concentrate, to divide one’s attention between multiple sensory events across visual and auditory modalities, and to make fast cognitive decisions in a complex and rapidly changing environment. Neuroimaging studies of neurophysiological variables have also elucidated the neural substrates involved during driving while performing sub-tasks such as conversation, auditory language comprehension, and visual event detection [5, 8, 9, 11, 14, 15, 17].

Several studies have been carried out to observe the changes in brain activation related to visual cognition [3–6], spatial attention or vigilance [3, 5], and motor function [14] while driving and performing sub-tasks simultaneously. It is reported that when performing driving and sub-tasks simultaneously, the activation of the parietal and occipital areas related to driving is reduced [8, 9, 11, 15]. It is also reported that when performing driving and sub-tasks simultaneously, the activation of the precentral gyrus, the frontal and parietal lobes, and cingulate gyrus areas related to attention, stimulus processing, motor responses, and decision-making is increased [5, 8, 9, 11, 14, 15, 17]. When performing driving and sub-tasks simultaneously, motor areas have decreased activation while the activation of areas related to sub-tasks, such as motor control and attention areas, is increased [8, 9, 11, 15].

However, the above studies were not realistic because the subjects performed the driving task using a joystick, computer mouses, or trackball with one hand in the simulated driving conditions (e.g., video game and driving simulator). In fact, driving is performed using a wheel (handle) and pedals. Therefore, it is difficult to determine the regions of the brain that are activated during the various cognitive activities required in actual driving using the systems used in previous studies. For this reason, some previous studies have attempted to simulate driving in a real driving environment by using a wheel and pedals. When using a computer mouse or a trackball to simulate driving and the sub-tasks simultaneously, fine control is actually worse than when a wheel and pedal is used in actual driving. For example, one can more accurately maintain a lane when controlling a wheel with both hands than when using a computer mouse or a trackball. In addition, one can more accurately control speed using the brake and one can more accurately control acceleration using the right foot than when using a computer mouse and a trackball. We, therefore, performed simulated driving in our study using a wheel and pedals to more accurately simulate driving control and the brain activation patterns present in an actual driving environment.

In order to overcome the limitations of previous studies, we used an MR-compatible driving simulator with a driving wheel and pedals in order to observe the effects of sub-tasks on driving. Our objective was to observe differences in activated brain regions using neurophysiological assessments during driving alone and when sub-tasks were performed during driving. Our working hypotheses were as follows. First, it is expected that the activation of the parietal area, which is the spatial perception-related area, will decrease and that the activation of areas related to sub-task performance will increase when performing driving and the sub-task simultaneously compared to driving only. Second, when performing driving and the sub-tasks simultaneously, the sub-tasks will affect driving. Therefore, the activation of areas related to behavior and motions used to control driving are expected to increase. Third, as previous studies simulated driving by using one hand (computer mouse, trackball, etc.), while this study used a wheel controlled with both hands and pedals controlled with the right foot, an additional area related to these actions is expected to be activated.

## Methods

Using the MR-compatible driving simulator for cases in which the driver only drives at 80 km/h (driving only), those in which the driver only performs the sub-task (task only), and those in which the driver performs the sub-task while driving at 80 km/h (driving with task), the research team designed a method to observe the brain using fMRI. In order to do this, we used subtraction and double subtraction methods.

### Subjects

Fifteen men with a driving experience of 2.5 ± 1.6 years and without any psychiatric illness or nerve/brain-related conditions were selected. Their average age was 26.0 ± 1.4 years. All subjects were right-handed as evaluated using the revised Edinburgh test [18]. Any subjects who might have had claustrophobia, pacemakers, or metal embedded in their bodies, which would have affected MR imaging, were excluded from the selection process. Prior to the experiment, all participants were prohibited from smoking, drinking alcohol/coffee, or any external activity that could impair their driving. The experiment was then explained to them. The subjects were required to practice in the simulation environment with the simulator until they could drive normally without crashing. The protocol for the research project was approved by the Institutional Review Committee of Konkuk University, where the work was undertaken. Our research protocol also conforms to the provisions of the Declaration of Helsinki (KU-IRB-11-46-A-1).

### MR-compatible driving simulator

**Fig. 1:**
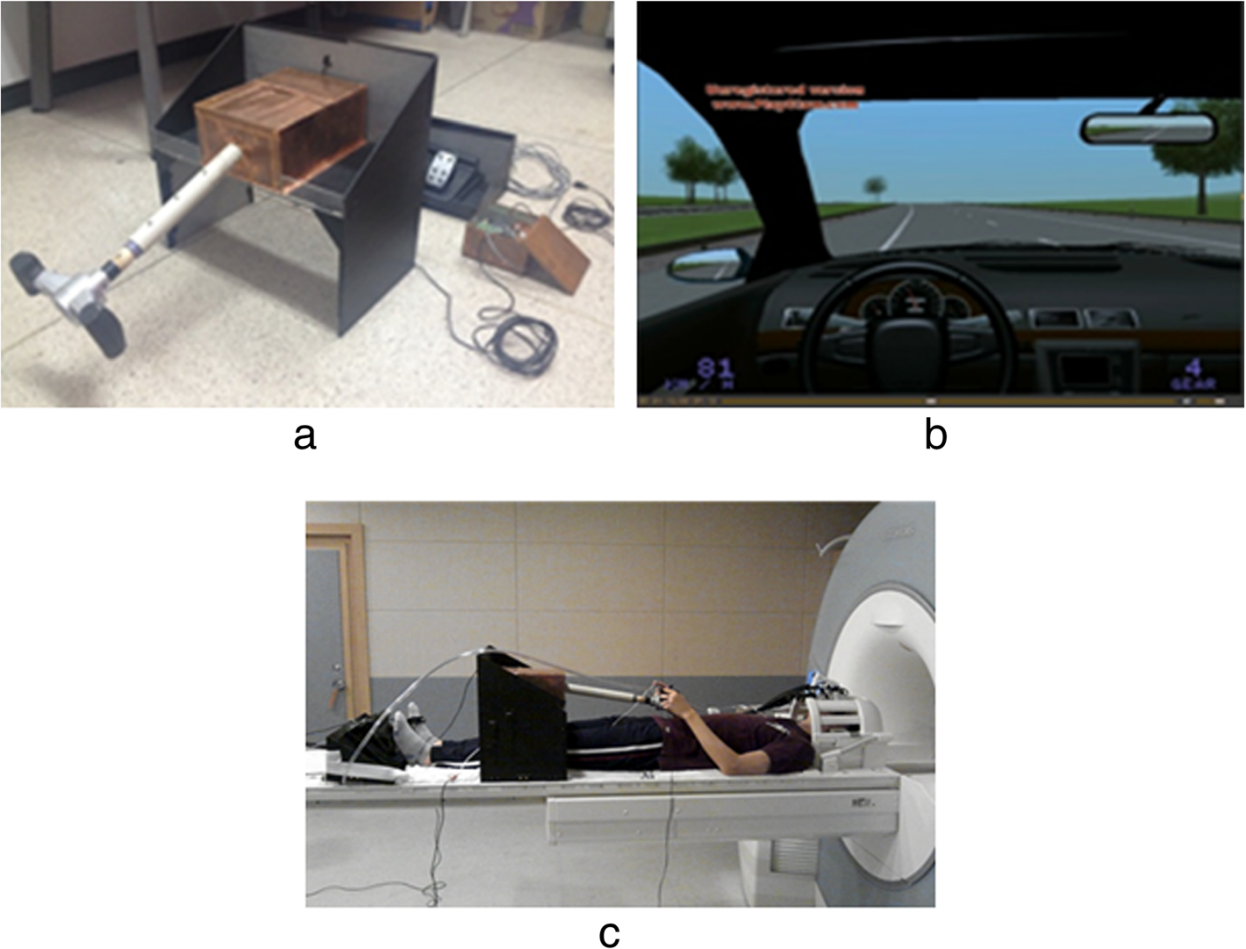
**a** MR-compatible driving simulator consisting of driving wheel, accelerator, and brake. **b** Driving environment (Lightrock Entertainment Inc., S/W). **c** Preparation before the experiment

The research team developed an MR-compatible driving simulator with a driving wheel and pedals, as seen in Fig. 1a. The simple driving environment was produced using software provided by Lightrock Entertainment, and was made up of mostly straight roadways with very few elements that could distract the driver (Fig. 1b). The subjects used both hands to operate the wheel and their right foot to control the accelerator and the brake. They drove at a constant 80 km/h without changing road lanes. Generally, in Korea, the speed limits on the roads are in accordance with Article 19 of “Road Traffic Act enforcement regulations” (speed of cars, etc.) and the highest speed limit on general roads with two or more one-way lanes (all roads other than highways and motorways) is less than 80 km/h. In consideration of safety and accessibility when preparing the test video, this study used general roads of two or more one-way lanes as the video, which was presented at a simulated speed of 80 km/h, which is the regulated speed limit on general roads. The visual information for driving was displayed on a visual system attached to the subjects’ head coil. Fig. 1c shows the preparation before the beginning of the experiment.

### Experimental design

**Fig. 2:**
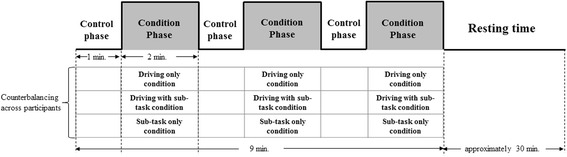
The functional magnetic resonance imaging (fMRI) experimental procedure

The experiment was performed using three conditions. Each condition consisted of three same blocks and every block has a control phase (1 min) and a condition phase (2 min). The first condition is when only driving is performed. In this condition, one block consists of a control phase (1 min) and a driving only condition (2 min) (Fig. 2a). The second condition is when driving and a sub-task are performed simultaneously. In this condition, one block consists of a control phase (1 min) and a driving with sub-task condition (2 min) (Fig. 2a). The third condition is when only a sub-task is performed. In this condition, one block consists of a control phase (1 min) and a sub-task only condition (2 min) (Fig. 2b). Commonly, during the control phase, the drivers were instructed to simply look at the screen, which showed a parked, non-moving state. During the driving only condition, the subjects were asked to only drive at 80 km/h. During the driving with sub-task condition, the drivers were asked to drive at 80 km/h while performing a sub-task. Finally, all subjects were asked to only perform a sub-task under identical conditions during the sub-task only condition. Each subject participated in the above three conditions (driving only, driving with sub-task, and sub-task only conditions). Once the first experiment was concluded, the subjects were all provided with plenty of resting time (approximately 30 min) and were then sent to the next experiment. The condition order was counterbalanced across participants. The speed of the vehicle was displayed on the lower left-hand corner of each subject’s screen in order to help them maintain a speed of 80 km/h.

The sub-task consisted of performing an addition. The addition task consisted of problems using double-digit numbers with sums of less than 100 and required carry-over calculations. Each block consisted of 10 problems. Thus, there were a total of 30 problems in the addition task. The experimenters used the audio system attached to the MR system to vocally present the task and the subjects confirmed the answers using their voices. The subjects were encouraged to concentrate on both driving and the addition tasks.

### Image acquisition

Images were scanned using a 3T MRI system (Magnetom TrioTim, Siemens Medical Systems, Erlangen, Germany) with a standard 32-channel head coil. Single-shot echo planar fMRI scans were acquired in 29 continuous slices parallel to the anterior commissure-posterior commissure line. The parameters for fMRI were as follows: repetition time (TR)/echo time (TE) = 3000/30 ms, field of view (FOV) = 200 mm, flip angle = 90°, matrix = 128 × 128, slice thickness = 5 mm, and voxel size = 1.6 × 1.6 × 5.0 mm. Anatomical images were obtained using a T1-weighted three-dimensional magnetization-prepared rapid gradient-echo sequence with TR/TE = 1900/2.48 ms, FOV = 200 mm, flip angle = 9°, matrix = 256 × 256, slice thickness = 1 mm, and voxel size = 0.8 × 0.8 × 1.0 mm.

### Image analysis

The fMRI data were analyzed using SPM 8 software (Wellcome Department of Cognitive Neurology, London, UK). All functional images were aligned with the anatomic images of the study using affine transformation routines built into the SPM 8 program. The realigned scans were co-registered to the participant’s anatomic images obtained within each session. The anatomical images were then segmented into white matter, gray matter, and cerebrospinal fluid. The mean echo planar image (EPI) of each subject was directly warped into the standard EPI template (Montreal Neurologic Institute) during a normalization step. The size of one divided voxel in normalizing process is 1.6 × 1.6 × 3 mm. The time-series data were motion-corrected by Sinc interpolation and filtered using a 240-s high-pass filter to remove artifacts because of cardiorespiratory and other cyclical influences. The functional images were then smoothed using a 8-mm full-width-half-maximum isotropic Gaussian kernel prior to statistical analyses. The statistical analysis was conducted using SPM 8 both individually (first level) and as a group (second level) using the general linear model and the theory of Gaussian random fields. Statistical parametric maps were computed using *t*-statistics. Individual subjects were analyzed at a significance threshold of *p* < 0.05, which was corrected using the topological peak-false discovery rate (FDR).

The active regions of the brain during driving only condition and driving with sub-task condition were extracted and compared to those of the control phase using the subtraction method ([driving only or driving with sub-task condition]—control). The double subtraction method was used to observe any regions exhibiting special activity during either driving condition (driving only—driving with sub-task and driving with sub-task—driving only conditions).

We also extracted the numbers of activation area voxels during driving only condition and driving with sub-task condition using the subtraction method for each subject. We then performed a paired *t*-test (PASW Statistics 18) to compare the numbers of activation area voxels by condition.

## Results

### Accuracy rate

When only the addition task was performed (sub-task only condition), the accuracy rate of the subjects was 84.8 ± 10.9%. The accuracy rate was 78.5 ± 11.7% when the addition task was combined with driving (driving with sub-task condition) (Table 1). No differences of note were observed using a paired *t*-test (PASW Statistics 18) (*p* = 0.196).

**Table 1:** Mean ± S.D. of the accuracy rate [%] results by every subject

Subject	Driving with sub-task condition	Sub-task only condition
#1	62.67	77.00
#2	84.67	74.28
#3	76.33	91.33
#4	88.00	90.67
#5	87.33	93.33
#6	89.00	94.45
#7	93.33	91.87
#8	70.00	58.23
#9	58.67	95.23
#10	63.88	90.52
#11	72.28	89.24
#12	85.74	94.11
#13	89.67	70.00
#14	64.56	73.25
#15	91.67	88.67
Mean ± S.D.	78.5 ± 11.7	84.8 ± 10.9

### Brain activation regions determined using the subtraction method

**Fig. 3:**
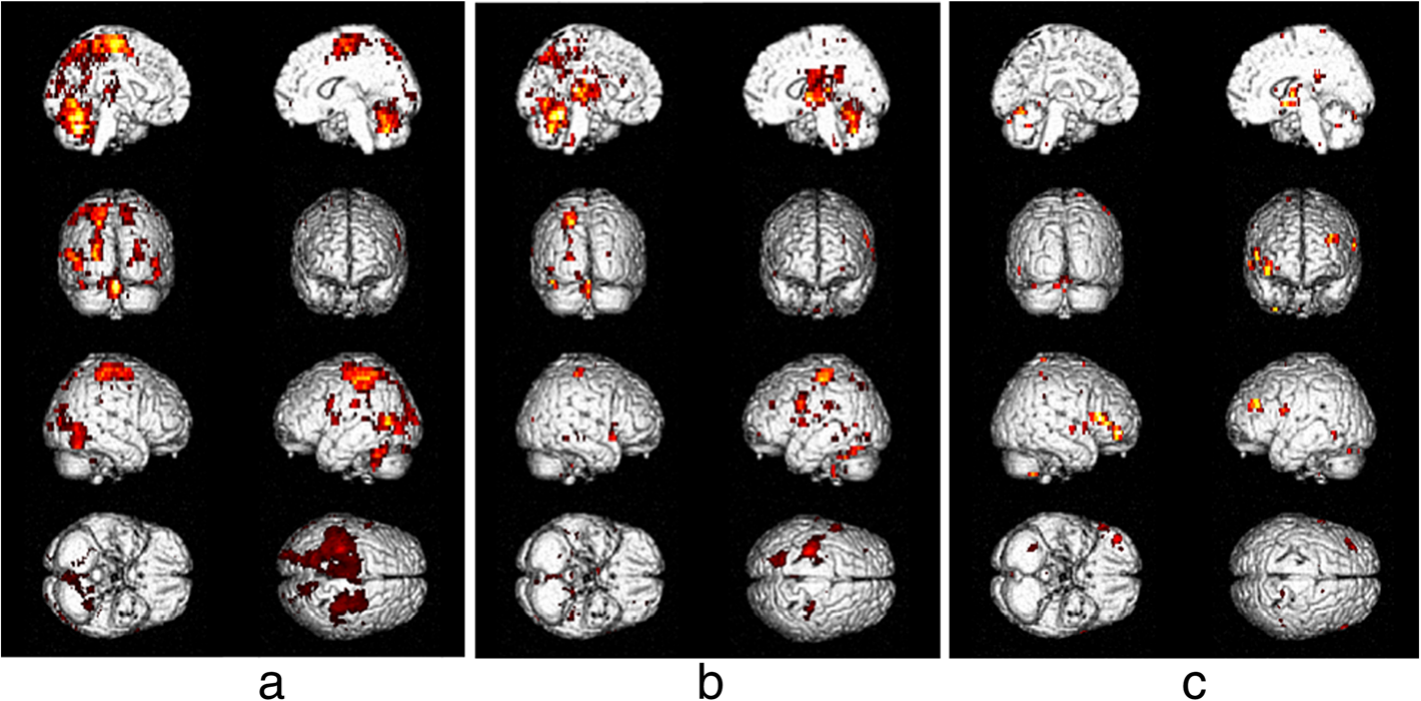
Brain activation areas for **a** driving only condition—control, **b** driving with sub-task condition—control, and **c** sub-task only condition—control (corrected *p* < 0.05 s)

During driving only condition, the frontal region (inferior frontal gyrus (IFG), middle frontal gyrus (MFG), superior frontal gyrus (SFG), and precentral gyrus), parietal region (superior parietal lobe (SPL), inferior parietal lobe (IPL), postcentral gyrus, and precuneus), temporal region (superior temporal gyrus (STG) and middle temporal gyrus (MTG)), occipital region (inferior occipital gyrus (IOG), superior occipital gyrus (SOG), middle occipital gyrus (MOG), and lingual gyrus), limbic region (cingulate gyrus), sub-lobar region (insula and lentiform nucleus), and the cerebellum (uvular, declive, and cerebellar tonsil) all exhibit activation (Table 2 and Fig. 3a).

**Table 2:** The MNI coordinates, *t*-scores, and number of voxels in the activated areas by the subtraction

Number of voxels	*t*-score	MNI coordinates (*x*,*y*,*z* (mm))	Side	Region	Brodmann area
352	7.78	51 −62 −10	R	Middle occipital gyrus	37
330	8.2	37 −4 60	R	Middle frontal gyrus	6
327	8.28	33 −28 65	R	Postcentral gyrus	3
309	7.86	22 −6 65	R	Superior frontal gyrus	6
178	7.51	8 −57 65	R	Precuneus	7
188	7.49	16 −68 55	R	Superior parietal lobule	7
146	6.78	44 −61 0	R	Middle temporal gyrus	37
10	5.37	50 −34 30	R	Inferior parietal lobule	40
10	5.22	11 10 40	R	Cingulate gyrus	32
10	5.12	31 −67 5	R	Lingual gyrus	19
9	5.53	34 −75 25	R	Superior occipital gyrus	19
5	5	51 0 45	R	Precentral gyrus	6
3007	10.07	−30 −28 60	L	Precentral gyrus	4
2966	9.91	−28 −48 55	L	Inferior parietal lobule	40
58	7.66	−56 7 25	L	Inferior frontal gyrus	9
77	7.14	−51 0 5	L	Superior temporal gyrus	22
20	5.19	−44 −6 15	L	Insula	13
9	5.37	−31 −89 −10	L	Inferior occipital gyrus	18
4	4.87	−23 −4 15	L	Lentiform nucleus	
3043	10.19	0 −62 −30	RC	Uvula	
175	8.56	41 −62 −20	RC	Declive	
50	6.37	−12 −46 −45	LC	Cerebellar tonsil	

Method (driving only condition—control) (corrected *p <* 0.05)

*R* right cerebrum, *L* left cerebrum, *RC* right cerebellum, *LC* left cerebellum

During driving with sub-task condition, the frontal region (IFG, MFG, SFG, precentral gyrus, and sub-gyral), parietal region (postcentral gyrus), temporal region (STG and fusiform gyrus), occipital region (IOG and lingual gyrus), limbic region (cingulate gyrus), sub-lobar region (lentiform nucleus, insula, claustrum, thalamus, cuneus, and caudate), and the cerebellum (cerebellar tonsil and inferior semi-lunar lobule) were shown to have activation (Table 3 and Fig. 3b).

**Table 3:** The MNI coordinates, *t*-scores, and number of voxels in the activated areas by the subtraction method (driving with sub-task condition—control) (corrected *p <* 0.05)

Number of voxels	*t*-score	MNI coordinates (*x*,*y*,*z* (mm))	Side	Region	Brodmann area
1433	8.56	30 −32 20	R	Insula	13
285	7.72	9 −4 0	R	Lentiform nucleus	
66	6.31	33 5 −10	R	Inferior frontal gyrus	13
63	5.98	33 −26 60	R	Precentral gyrus	4
57	6.49	45 11 −10	R	Superior temporal gyrus	38
55	5.15	45 −32 60	R	Postcentral gyrus	2
54	5.24	34 −4 −5	R	Claustrum	
46	5.17	9 −25 20	R	Thalamus	
14	5.38	22 7 30	R	Cingulate gyrus	32
12	5.69	25 −82 10	R	Cuneus	17
11	5.87	50 −40 −10	R	Fusiform gyrus	37
1879	9.1	−28 −31 60	L	Postcentral gyrus	3
1791	8.72	−31 −40 25	L	Insula	13
156	7.95	−55 3 30	L	Precentral gyrus	6
59	5.66	−50 −1 5	L	Superior temporal gyrus	22
32	5.81	−11 −84 −10	L	Lingual gyrus	18
32	5.57	−28 19 10	L	Claustrum	
27	5.76	−42 −76 −5	L	Inferior occipital gyrus	19
24	5.41	−30 38 30	L	Middle frontal gyrus	9
20	5.93	−20 −81 25	L	Cuneus	18
19	5.09	−9 −3 25	L	Caudate	
8	5.36	−28 53 −5	L	Superior frontal gyrus	10
6	4.85	−25 −6 55	L	Sub-gyral	6
8	5.35	28 −36 −40	RC	Cerebellar tonsil	
1908	9.46	0 −59 −35	LC	Inferior semi-lunar lobule	

*R* right cerebrum, *L* left cerebrum, *RC* right cerebellum, *LC* left cerebellum

In order to compare the numbers of activation area voxels for each condition calculated using the above subtraction method, we performed a paired *t*-test by extracting the areas activated in common.

The number of activation voxels was significantly reduced in areas such as the MFG (*p* = 0.046) and the precentral gyrus (*p* = 0.033) during driving with sub-task condition when compared to the driving only condition. On the other hand, when driving and performing the sub-tasks simultaneously, the number of activation voxels was significantly increased in the postcentral gyrus (*p* = 0.031) and the insula (*p* = 0.011) when compared to driving only condition. Other commonly activated areas were IFG, SFG, STG, IOG, cingulate gyrus, lentiform nucleus, and the cerebellar tonsil. There were no significant differences between the two conditions in these areas.

During sub-task only condition, the frontal region (IFG, MFG, SFG, and precentral gyrus), parietal region (IPL), temporal region (inferior temporal gyrus (ITG), MTG, STG, and caudate), limbic region (cingulate gyrus), sub-lobar region (lentiform nucleus and insula), and the cerebellum (uvula, culmen, and declive) showed signs of activation (Table 4 and Fig. 3c).

**Table 4:** The MNI coordinates, *t*-scores, and number of voxels in the activated areas by the subtraction method (sub-task only condition—control) (corrected *p <* 0.05)

Number of voxels	*t*-score	MNI coordinates (*x*,*y*,*z* (mm))	Side	Region	Brodmann area
200	5.64	42 46 −5	R	Middle frontal gyrus	10
192	4.41	36 21 10	R	Insula	13
174	5.08	58 30 10	R	Inferior frontal gyrus	46
154	5.36	16 3 −5	R	Lentiform nucleus	
45	3.75	22 −34 30	R	Cingulate gyrus	31
26	3.94	61 −7 −5	R	Middle temporal gyrus	21
26	3.55	48 −43 60	R	Inferior parietal lobule	40
12	3.61	55 8 5	R	Precentral gyrus	44
11	3.26	59 7 −5	R	Superior temporal gyrus	22
6	3.66	17 7 70	R	Superior frontal gyrus	6
112	4.46	−31 41 25	L	Middle frontal gyrus	10
48	4.22	−59 5 20	L	Precentral gyrus	6
23	4.07	−30 −37 5	L	Caudate	
20	3.82	−51 −54 −5	L	Inferior temporal gyrus	37
7	3.28	−9 −3 65	L	Superior frontal gyrus	6
23	3.97	0 −61 −30	RC	Uvula	
12	3.72	2 −45 −5	RC	Culmen	
109	4.85	−5 −70 −15	LC	Declive	
11	3.4	−34 −71 −25	LC	Uvula	

*R* right cerebrum, *L* left cerebrum, *RC* right cerebellum, *LC* left cerebellum

### Brain activation regions determined using the double subtraction method

The double subtraction method was used to observe the regions of the brain that exhibited special activation when the subject was only driving (driving only condition) or driving while performing a sub-task (driving with sub-task condition).

**Fig. 4:**
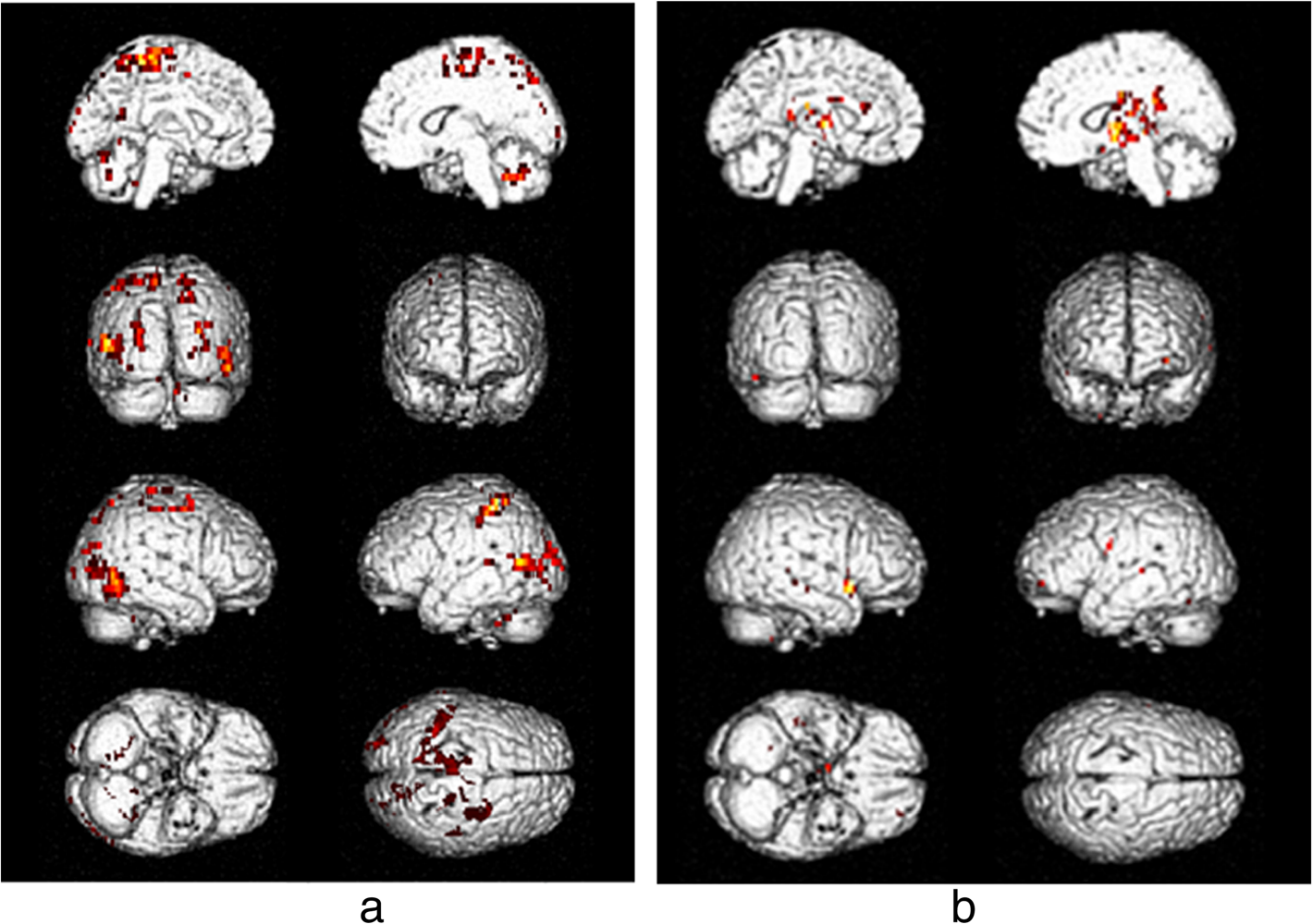
Brain activation areas for **a** driving only condition—driving with sub-task condition and **b** driving with sub-task condition—driving only condition (corrected *p* < 0.05)

The brain regions activated during driving only condition were subtracted from the regions that were activated during driving with sub-task condition. These results are presented in Table 5 and Fig. 4a. The results show that brain activation is increased in the frontal region (MFG, medial frontal gyrus (MeFG), and precentral gyrus), parietal region (SPL, IPL, postcentral gyrus, and precuneus), temporal region (MTG and STG), occipital region (IOG, SOG, MOG, lingual gyrus, and cuneus), limbic region (cingulate gyrus), and the cerebellum (uvula, declive, inferior semi-lunar lobule, and cerebellar tonsil). In particular, we observed a large increase in the superior parietal lobule. Large increases were also seen in the middle frontal gyrus, the middle occipital gyrus, and the uvula of the cerebellum. These regions correspond to those that have reduced or no activation when driving is performed along with secondary activities.

**Table 5:** The MNI coordinates, *t*-scores, and number of voxels in the activated areas by the double subtraction method (driving only condition—driving with sub-task condition) (corrected *p <* 0.05)

Number of voxels	*t*-score	MNI coordinates (*x*,*y*,*z* (mm))	Side	Region	Brodmann area
129	7.3	36 −3 60	R	Middle frontal gyrus	6
110	7.88	51 −62 −10	R	Middle occipital gyrus	37
82	6.59	45 −62 0	R	Middle temporal gyrus	37
80	7.08	23 −28 70	R	Postcentral gyrus	3
80	6.17	28 −87 20	R	Cuneus	19
62	6.4	17 −70 55	R	Superior parietal lobule	7
21	5.4	23 −20 70	R	Precentral gyrus	6
16	5.6	34 −75 25	R	Superior occipital gyrus	19
12	5.13	31 −67 5	R	Lingual gyrus	19
11	5.43	17 −64 40	R	Precuneus	7
8	6.42	9 −3 50	R	Cingulate gyrus	24
627	8.82	−30 −45 55	L	Superior parietal lobule	7
156	7.11	−23 −87 15	L	Middle occipital gyrus	19
75	7.36	−50 −65 10	L	Middle temporal gyrus	39
74	6.37	−66 −37 10	L	Superior temporal gyrus	22
22	6.19	−53 −32 45	L	Inferior parietal lobule	40
16	6.88	−5 2 50	L	Medial frontal gyrus	6
6	5.52	−16 −59 −5	L	Lingual gyrus	19
6	5.23	−31 −89 −10	L	Inferior occipital gyrus	18
125	8.28	8 −67 −30	RC	Uvula	
17	6.65	37 −64 −15	RC	Declive	
50	6.71	−11 −67 −35	LC	Inferior semi-lunar lobule	
25	6.23	−34 −46 −40	LC	Cerebellar tonsil	
19	6.2	−9 −71 −20	LC	Declive	

*R* right cerebrum, *L* left cerebrum, *RC* right cerebellum, *LC* left cerebellum

To determine the activated brain regions during driving with sub-task condition, the regional activation during driving with sub-task condition is subtracted from the activation during driving only condition. These results are summarized in Table 6 and Fig. 4b. The results indicate an increased activation in the frontal region (IFG, SFG, and precentral gyrus), temporal region (STG), limbic region (cingulate gyrus), sub-lobar region (lentiform nucleus, caudate, insula, and thalamus), and the cerebellum (culmen and declive). In particular, there are large activation increases in the superior temporal gyrus, the cingulate gyrus, the sub-lobar area, which includes the lentiform nucleus, caudate, insula, and thalamus.

**Table 6:** The MNI coordinates, *t*-scores, and number of voxels in the activated areas by the double subtraction method (driving with sub-task condition—driving only condition) (corrected *p <* 0.05)

Number of voxels	*t*-score	MNI coordinates (*x*,*y*,*z* (mm))	Side	Region	Brodmann area
145	7.72	9 −4 0	R	Lentiform nucleus	
118	7.29	31 −34 10	R	Caudate	
113	6.59	45 11 −10	R	Superior temporal gyrus	38
101	5.61	44 −28 −5	R	Insula	22
89	6.4	19 −39 25	R	Cingulate gyrus	31
61	6.03	2 −31 0	R	Thalamus	
58	6.49	33 7 −10	R	Inferior frontal gyrus	13
68	7.24	−14 −6 10	L	Thalamus	
16	5.14	−9 −3 25	L	Caudate	
16	5.31	−30 25 15	L	Insula	13
12	5.22	−55 −1 25	L	Precentral gyrus	6
7	5.71	−28 52 −5	L	Superior frontal gyrus	10
6	5.06	−62 −28 5	L	Superior temporal gyrus	22
15	5.78	2 −40 0	RC	Culmen	
8	6.53	−44 −68 −20	LC	Declive	

*R* right cerebrum, *L* left cerebrum, *RC* right cerebellum, *LC* left cerebellum

## Discussion

Here we analyzed the effects of conducting a sub-task (addition task) during driving on brain activation using fMRI.

Notable differences were not observed in the accuracy rates sub-task only condition vs. driving with sub-task condition. It was expected that all of the experiment participants would show a higher task performance ability in the sub-task only condition rather than the driving with sub-task condition. Of the total of 15 participants, however, three showed opposite results. Before the experiment, all of the participants were requested to concentrate on the experiment (driving, task performance, etc.). But the three participants are deemed to have low concentration during their task performance in the sub-task only condition to show such a low accuracy. Additional study will be necessary in this regard.

Brain activation during driving situations has consistently been the subject of ongoing research [3–17]. Just et al. [9] used a computer mouse and trackball to simulate driving and reported that the parietal cortex, occipital cortex, motor cortex, and the cerebellum exhibit activation during this task. Uchiyama et al. [15] used a joystick to control the vehicles and observed the IFG, MFG, SFG, IPL, SPL, MTG, basal ganglia, primary sensorimotor cortex (S1), and SMA. Hsieh et al. [7] noted that while a driving scene is simply being observed, the MFG, IFG, STG, orbitofrontal cortex, occipital lobe, fusiform gyrus, cingulate gyrus, SMA, and the basal ganglia show activation. The results that we obtained during driving only condition show similar areas of activation (motor cortex, IPL, SPL, fusiform gyrus, and cerebellum; Table 2) to those observed in previous research. Previous studies [7, 9, 15] simulated driving using a computer mouse or trackballs. In these studies, one hand was used in the simulator environment. However, in this study, the subjects controlled a driving wheel using both hands in a real driving environment and controlled pedals using the ankle of the right lower limb. Because of the movement of both the hands and the right foot, the left precentral gyrus (primary motor cortex, M1) appeared to have the voxels with the highest activation. We also observed activation of the motor control-related right premotor cortex, which is used in adjusting precise movements [5, 13]. Since tactile sensory feedback is also generated through the wheel and the pedals, the right postcentral gyrus (S1) was activated [15, 19]. The activation of the somatosensory association cortex in the left IPL (BA 40) at the supramarginal gyrus [19] was especially great. In addition, we observed activations of the right precuneus area, which is related to visuomotor coordination [5, 12], and the right fusiform gyrus (BA 37), which is related to high-level visual cognition [7]. We also observed a larger number of activation voxels in the uvula of cerebellum, which is an area related to motor control and action planning [3, 4, 15].

When driving and a secondary task (auditory language comprehension task) were performed simultaneously, similar areas (motor cortex and the parietal and occipital lobes) were activated. This is similar to what is observed when only driving is performed. However, the numbers of activation voxels and activity intensity was decreased in these areas while the temporal and inferior frontal regions related to the sub-task had increases in activation [9]. Uchiyama et al. [15] observed the activation of similar areas (motor cortex and parietal and occipital lobes) when driving was performed along with an auditory task. However, they also reported activation of the STG and the primary auditory cortex. We observed that similar regions (motor cortex, SPG, IPG, and MOG) were activated to those activated when the subjects only drove (Table 3). However, the numbers of activation voxels of these regions were decreased and the IFG and STG, which are related to the addition task showed activation. The IFG was noted as an area significantly associated with the performance of additional tasks in previous research [20, 21]. Here, the same areas were activated during driving with sub-task condition and sub-task only condition (Tables 3 and 4). The additional activation of the STG is thought to occur because the experiment used in the study required the subjects to listen to and then respond to the additional task (Tables 3 and 4). Unlike in previous studies [9, 15], we observed that the number of activation voxels in the postcentral gyrus (S1) and the insular cortex appeared high in driving with sub-task condition. Unlike previous studies, where a joystick, computer mouse, or trackball was used with one hand, our study required the subjects to adjust a driving wheel with both hands and pedals with the right foot. The grip force of both hands required to hold the driving wheel to concentrate (lane keeping) on driving increased when driving with sub-task condition compared to driving only condition. In addition, the right foot touched the pedal more frequently to keep the speed at 80 km/h. Thus, the left postcentral gyrus area, which is the somatosensory area [19, 22], is expected to be activated significantly. In addition, when driving is performed simultaneously with a sub-task, hand movements were used to finely control the wheel in order to maintain the lane. These movements and those of the right ankle use for controlling the pedal to keep the speed at 80 km/h were more frequent. Thus, activation of the insula, which is related to perception, motor control, self-awareness, and cognitive function [23], was shown to be significantly increased in both hemispheres.

Just et al. [9] used the double subtraction method to analyze differences in brain activation while driving and driving while performing a secondary task (an auditory language comprehension task). When drivers were focused only on driving, compared to when they drove while performing an auditory distraction task of language comprehension, the supramarginal gyrus, SPL, IPL, and SOG showed increased activation. In particular, activation voxels of the SPL had the largest increase. Based on our results, when drivers were focused only on driving compared to when they drove while performing a task, the SPL and IPL areas, which are related to spatial perception [24], showed increased activation, similar to observations in previous studies. The visuomotor coordination-related left SPL [5, 12] showed the largest increase.

When drivers were focused only on driving compared to when they drove while performing a task, activation is thought to have increased in the above areas because the subjects were focused only on driving while controlling the wheel with both hands and controlling the pedals with their right foot. The parietal area is in charge of not only spatial processing but also visual spatial attention [24]. Thus, it has been determined that secondary activities negatively affect functions related to driving. Unlike previous studies [9], our study indicates that when drivers are focused driving only condition compared to driving with sub-task condition, the right MFG, which is the premotor cortex area related to spatial attention, movement planning, and execution [5, 13], the visual-related right and left MOG [3], and the action planning and motor control-related uvula of the cerebellum [3, 4, 15] show remarkably increased activation (Table 5). The negative effects on these areas are thought to occur because performing sub-tasks interferes with information processing during driving.

Compared to cases where only driving was performed, driving while performing additional tasks was shown to decrease brain activation in some regions, whereas other regions related to activity completion were additionally activated [7, 9, 15]. We observed that driving with sub-task condition was performed compared to driving only condition, the SPL, MFG, MOG, and cerebellar regions related to driving had less activation (Table 5). In contrast, there was increased activation in the IFG and the STG, which affect secondary task completion (Tables 3, 4, and 6). The cingulate gyrus region and the sub-lobar region (lentiform nucleus, caudate, insula, and thalamus) in particular showed evidence of increased activation (Table 6). These results can be attributed to the fact that when driving is paired with a secondary task, driving performance is affected. Moreover, the increase in desire to control increases the activation of the cingulate gyrus and sub-lobar regions, as they control error monitoring and unnecessary movement control, respectively [5, 13, 23, 25]. In addition, during driving with sub-task condition, we observed activation of the right temporal gyrus (BA 38) region, which is related to highly processed perceptual inputs to visceral responses, for complex cognitive processing [7, 15].

### Limitation

Although not performed in this study, it is necessary to analyze the relationship between actual driving performance and brain activity by additionally extracting the activation patterns for a variety of driving performance conditions (maintaining driving speed, responding to uncertainties during driving, lane keeping, etc.) when driving and the sub-task are performed simultaneously. We would then carry out a study on how the sub-task affects actual driving performance.

## Conclusions

In conclusion, when driving and a sub-task were performed together (driving with sub-task condition—driving only condition), the regions associated with driving show less activation, as observed in previous studies. In particular, the spatial perception regions have the largest decreases among all the regions assessed. Unlike in previous research [7, 9, 15], our double subtraction results (driving with sub-task condition—driving only condition) indicate a clear increase in activation in the cingulate gyrus and the sub-lobar region. Unlike the simple driving simulators used in previous research, which used joysticks, computer mouses, or trackballs for simulation, the addition of a driving wheel and pedals (accelerator and brake) to the driving simulator used in this study closely represents real driving. Thus, the number of processed movements increases, leading to an increased number of unnecessary movements that need to be controlled. This in turn increases the activation of the corresponding brain regions.

There are numerous research studies investigating brain activation changes using simple driving simulators equipped with joysticks, computer mouses, or trackballs. However, research studies similar to ours, which employs a simulator that closely reflects reality, have been sparse in comparison. Further research on the effects of sub-tasks on brain activation of drivers of varied driving skills is required. This paper is expected to contribute basic data toward studying the effects of sub-tasks during driving.
